# Characterisation of Plasmid-Associated Antimicrobial Resistance Genes in Coastal Marine *Enterobacterales* from the Central Adriatic Sea: De Novo Assembly and Bioinformatic Profiling

**DOI:** 10.3390/ijms262210910

**Published:** 2025-11-11

**Authors:** Ivica Šamanić, Mia Dželalija, Ema Bellulovich, Hrvoje Kalinić, Slaven Jozić, Marin Ordulj, Nikolina Udiković-Kolić, Ana Maravić

**Affiliations:** 1 Department of Biology, Faculty of Science, University of Split, 21000 Split, Croatia; mdzelalij@pmfst.hr; 2 Center for Proteomics, Faculty of Medicine, University of Rijeka, 51000 Rijeka, Croatia; ema.bellulovich@medri.uniri.hr; 3 Department of Informatics, Faculty of Science, University of Split, 21000 Split, Croatia; hrvoje.kalinic@pmfst.hr; 4 Laboratory of Marine Microbiology, Institute of Oceanography and Fisheries, 21000 Split, Croatia; sjozic@izor.hr; 5 Department of Marine Studies, University of Split, 21000 Split, Croatia; mordulj@unist.hr; 6 Division for Marine and Environmental Research, Ruđer Bošković Institute, 10000 Zagreb, Croatia; nudikov@irb.hr

**Keywords:** plasmid-associated resistance, coastal *Enterobacterales*, Adriatic Sea, mobile genetic elements, replicon typing, One Health

## Abstract

This study examines the genomic composition and resistance potential of eight putative plasmid-derived contig assemblies reconstructed from marine *Enterobacterales* isolated in the central Adriatic Sea. Using a combination of Illumina-based whole genome sequencing, de novo assembly, and a multi-tool bioinformatics pipeline, we annotated antimicrobial resistance genes (ARGs), insertion sequences (ISs), and plasmid replicon types. Clinically significant resistance markers such as *bla*_KPC_, *bla*_TEM_, *aacA4*, *tetA*, and *folP* were identified, frequently co-localised with mobile genetic elements including IS110, IS4, and IS1182. The plasmid-associated contigs were assigned to MOBP and MOBQ types and contained replicon markers (IncP6, IncA/C2) characteristic of broad-host-range plasmids. Our findings provide valuable insight into the plasmidome of environmental *Enterobacterales*, emphasising the role of coastal pollution in shaping the distribution and potential mobility of antimicrobial resistance genes. This supports the One Health framework by linking environmental reservoirs to clinically relevant resistance mechanisms.

## 1. Introduction

The global increase in antibiotic resistance genes (ARGs) represents one of the greatest threats to public health in the 21st century, affecting both the clinical and environmental microbiome [1]. Among mobile genetic elements, plasmids play a central role as vectors of ARGs, enabling their rapid spread across bacterial populations and even species boundaries [2]. Plasmids often carry not only resistance determinants, but also insertion sequences (IS), transposons, integrons, and conjugative elements that contribute to their horizontal transmission capability [3].

Marine and coastal environments are increasingly recognised as reservoirs and mixing grounds for ARGs, especially in regions affected by anthropogenic activities such as sewage discharges, aquaculture, and maritime traffic [4]. In these systems, members of the order *Enterobacterales*—particularly *Escherichia coli*, *Enterobacter* spp., and *Klebsiella pneumoniae*—can persist in water or sediments and act as environmental carriers of resistance genes [5,6]. These bacteria are also important pathogens in humans, frequently involved in bloodstream infections, urinary tract infections, and hospital-acquired pneumonia [7]. Their detection in coastal waters indicates fecal contamination and is a possible route of transmission from the environment to humans [8]. The central Adriatic coast, especially near urban areas, is a high-risk interface where municipal wastewater, hospital effluents, and stormwater runoff may contribute to the marine resistome [9,10]. This highlights the importance of examining ARG dissemination in environments where human activities intersect with vulnerable coastal ecosystems.

Of particular concern is the spread of multidrug resistance plasmids, many of which encode carbapenemases (e.g., *bla*_KPC_, *bla*_VIM_), aminoglycoside-modifying enzymes and tetracycline or sulfonamide resistance genes. The co-localisation of these genes with virulence factors and mobile elements increases the epidemiological risk of such plasmids [11].

Despite their importance, ARG-carrying plasmids in marine *Enterobacteria* are still poorly characterised. Previous studies in the Adriatic Sea have identified clinically relevant ARGs in wastewater, seawater, and sediments using metagenomic and qPCR-based methods, but without plasmid-level resolution or structural characterisation of ARG-carrying elements, which limited inferences about their mobility and dissemination potential [10,12,13].

In this study, we investigated the structure, function, and mobility of putative plasmid-derived assemblies isolated from marine *Enterobacterales* collected from seawater samples near an urban and hospital-influenced coastal site in the central Adriatic Sea. Using short-read whole-genome sequencing, de novo assembly, and a suite of complementary functional annotation tools (including Prokka v1.14.6+galaxy1 [14], MOB-suite v3.1.0+galaxy0 [15], ABRicate v1.0.1+galaxy0 [16] with the CARD v4.0.2 and ResFinder v4.4.2 databases [17], and ISEScan v1.7.2.3+galaxy0 [18]), we achieved a comprehensive characterisation of antimicrobial resistance genes (ARGs) and associated mobile genetic elements. Our results provide new insights into the plasmidome of *Enterobacterales* in coastal areas and their potential contribution to environmental reservoirs of resistance. This work expands our understanding of ARG circulation at the land–sea interface and emphasises the importance of plasmid epidemiology for marine public health surveillance within the One Health framework.

## 2. Results

### 2.1. Characterisation of Reconstructed Plasmid-like Sequences from Marine Enterobacterales

#### 2.1.1. Reconstruction and General Characteristics of the Predicted Plasmids

**Figure 1:**
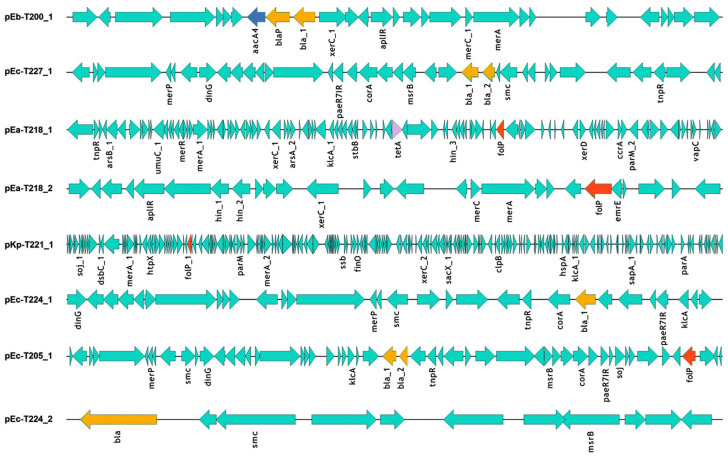
Linear genetic maps of reconstructed plasmid-like assemblies from marine *Enterobacterales* isolates. Each plasmid sequence is shown linearly, with the orientation of the genes indicated by the direction of the arrow. Annotated features include antibiotic resistance genes (e.g., *bla*, *tetA*, *aacA4*, *folP*), virulence factors (e.g., *vapC*), mobile elements (e.g., *tnpR*, mer operons) and conserved backbone genes. The maps were generated with EasyFig [19] based on Prokka-annotated .gbk files [14]. ARGs are colour-coded by resistance class: *bla* genes are shown in yellow, *aacA4* in blue, *tetA* in purple, *folP* in red, and all other annotated genes in green.

Eight putative plasmid-like contig clusters were reconstructed from Illumina short-read data using de novo assembly and plasmid classification pipelines. These assemblies originated from *Enterobacterales* isolates obtained from central Adriatic coastal seawater. The MOB-suite mob-recon module [15] was used to infer plasmid-associated contigs, revealing considerable variation in plasmid size and gene content across samples (Figure 1; Supplementary Figures S1–S8). The largest contig cluster, pKp-T221_1 (~163,845 bp total length across 85 contigs), was derived from *K. pneumoniae*. Other assemblies ranged from ~7.5 to ~43 kb in total length and were associated with *E. coli*, *E. bugandensis* and *E. asburiae* strains. The results represent putative reconstructions based on sequence signatures (e.g., replicon and MOB genes), rather than circularised plasmid sequences. The linear maps generated using EasyFig v2.2.5 [19] illustrate the arrangement of predicted resistance genes, mobility elements, and conserved functional modules (Figure 1). Annotated regions included backbone genes such as mer operons, *tnpR*, and *dinG*, which were observed in several isolates, indicating conserved functional elements potentially important for plasmid maintenance and environmental persistence.

#### 2.1.2. Antibiotic Resistance Genes (ARGs)

Nine distinct antibiotic resistance genes (ARGs) were identified across the eight reconstructed plasmid-like assemblies using the CARD [16], ResFinder [17] and NCBI databases (Table 1). These ARGs conferred resistance to five antibiotic classes: β-lactams, aminoglycosides, sulfonamides, tetracyclines, and trimethoprim, indicating broad-spectrum resistance potential among *Enterobacterales* isolates from the central Adriatic coastal environment. β-lactamase genes were the most prevalent and clinically relevant resistance determinants. The *bla*_KPC_ gene, which encodes a class A carbapenemase, was identified in four *E. coli* plasmid-like contig clusters (pEc-T224_1, pEc-T224_2, pEc-T227_1, and pEc-T205_1). This gene is known for its role in carbapenem hydrolysis and was frequently co-detected with *bla*_TEM_, a broad-spectrum β-lactamase associated with resistance to penicillins and early cephalosporins (pEc-T227_1, pEc-T205_1). Additionally, *bla*_GES_ (an extended-spectrum β-lactamase capable of hydrolysing cephalosporins and occasionally carbapenems) and *bla*_OXA_ (a class D oxacillinase) were co-localised on pEb-T200_1 (*E. bugandensis*), forming a multidrug-resistant gene cluster.

Aminoglycoside resistance genes included *aacA4*, which encodes an acetyltransferase that modifies the 6′-amine group of aminoglycosides, conferring resistance to gentamicin, tobramycin, and netilmicin. This gene was present on pEa-T218_1 (*E. asburiae*) and pEb-T200_1 (*E. bugandensis*). Additionally, *ant(3″)-Ia* (also annotated as *ant1*), a streptomycin adenylyltransferase, was detected on pKp-T221_1 (*K. pneumoniae*), indicating that aminoglycoside-modifying enzymes are widely distributed across species and assembly types.

Sulfonamide resistance was mediated by *folP*, which encodes a variant of dihydropteroate synthase that reduces sulfonamide binding. Notably, *folP* was identified in three host species (*E. asburiae*, *K. pneumoniae*, *E. coli*) and four plasmid-like assemblies (pEa-T218_1, pEa-T218_2, pKp-T221_1, pEc-T205_1), supporting its stability and potential for horizontal transfer between taxa.

Trimethoprim resistance was conferred by *dfrB1*, which encodes a drug-insensitive dihydrofolate reductase. This gene was exclusive to pKp-T221_1, where it co-occurred with *folP* and *ant1*, suggesting the formation of a composite resistance island.

Tetracycline resistance was conferred by *tetA*, which encodes an efflux pump that reduces intracellular tetracycline concentration. This gene was found in pEa-T218_1, contributing to the multidrug-resistance profile of *E. asburiae* in the coastal setting.

Most ARGs were co-localised with insertion sequences (ISs) such as IS110, IS6, IS4, and IS1182, and associated with replicon types IncP6 and IncA/C2, as well as MOB types MOBP and MOBQ, implying mobility and a risk of horizontal dissemination (Table 2). These features reflect the structural modularity of environmental plasmid-derived elements, contributing to the persistence and spread of resistance in marine ecosystems under anthropogenic influence.

#### 2.1.3. Mobile Elements and Structural Features

The insertion sequences were widely distributed across the plasmid-like assemblies, with IS110, IS1182, IS4, and IS6 being the most abundant (Table 2). The diversity of IS elements was greatest on the largest assembly, pKp-T221_1, which also carried composite structures such as ISNCY and ISL3. These results suggest an increased capacity for horizontal gene transfer and structural rearrangements in marine environments. While insertion sequences were detected across many contigs, the structural organisation of complete transposable units remains unresolved due to assembly fragmentation inherent to short-read data. Mobility predictions classified most plasmids as MOBP type, with the exception of pEb-T200_1, which was identified as MOBQ (Table 2). The MOBP replicon type was consistently associated with the *bla*_KPC_-carrying plasmid-like assemblies. Incompatibility (Inc) typing via PlasmidFinder (v2.1.1) [21] showed the dominance of IncP6 and IncA/C2 backbones among the larger assemblies (Table 2). Notably, pKp-T221_1 was the only sequence assigned to IncA/C2, consistent with its larger size and higher density of IS elements. These classifications provide indirect evidence of mobilisation potential, but actual conjugative transfer cannot be inferred without functional validation or long-read confirmation of circularity.

#### 2.1.4. Virulence Determinants

The content of virulence genes was limited. The *astA* gene, which encodes a heat-stable enteroaggregative *E. coli* enterotoxin, was identified in pEa-T218_1, while pKp-T221_1 was annotated with *clpK2*, a heat shock protein gene. The *clpK2* annotation likely corresponds to *clpB*, a gene encoding a heat-shock chaperone associated with general stress tolerance rather than virulence. No additional virulence determinants were detected. The overall scarcity of classical virulence genes in these plasmid-like assemblies supports the notion that environmental *Enterobacterales* plasmids primarily function as resistance vectors rather than direct contributors to pathogenicity.

### 2.2. Comparative Alignment and ARG Mapping

**Figure 2:**
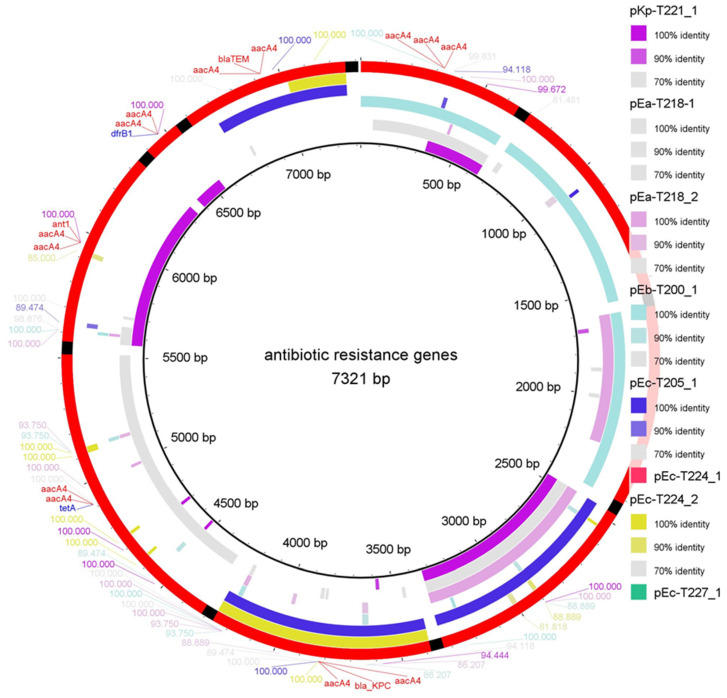
Comparative mapping of antibiotic resistance genes in de novo assembled plasmid-like sequences using BRIG. BLAST-based alignment of a curated ARG reference set (total length: 7321 bp) against reconstructed plasmid assemblies. The outermost ring represents the multi-FASTA reference of the ARGs (e.g., *aacA4*, *bla*_KPC_, *bla*_TEM_, *dfrB1*, *tetA*, and *ant1*). The concentric inner rings correspond to the assemblies (.fna files) of pKp-T221_1, pEa-T218_1, pEa-T218_2, pEb-T200_1, pEc-T205_1, pEc-T224_1, pEc-T224_2, and pEc-T227_1. Colour gradients reflect BLAST identity (100%, 90%, 70%). The visualisation was created using BLAST+ in BRIG (v0.95) [22] to assess ARG presence and sequence conservation.

To assess the distribution and conservation of major antibiotic resistance genes (ARGs) among the de novo assembled plasmid-like sequences, a reference set of ARGs was aligned to the reconstructed assemblies using BLAST in BRIG v0.95 [22] (Figure 2). The putative plasmid-derived contigs pEa-T218_1, pEb-T200_1, and pEc-T205_1 show notably high ARG content. The plasmid-like assemblies pEc-T224_1 and pEc-T227_1 both harbour *bla*_KPC_, as indicated by a clear alignment at 100% identity near the 4000–5000 bp region of the ARG reference sequence. In contrast, pEc-T224_2 and pEa-T218_2 show only minimal or fragmented alignment with the ARG reference set, suggesting limited resistance gene content. These results highlight the heterogeneity of the ARG distribution among the reconstructed assemblies of different *Enterobacterales* strains, with *bla*_KPC_ identified as the most frequently detected resistance determinant. The colour gradations indicate different degrees of sequence conservation (Figure 2), with most alignments falling in the 90–100% identity range, while lower identity regions likely correspond to partial gene sequences or flanking mobile elements.

**Figure 3:**
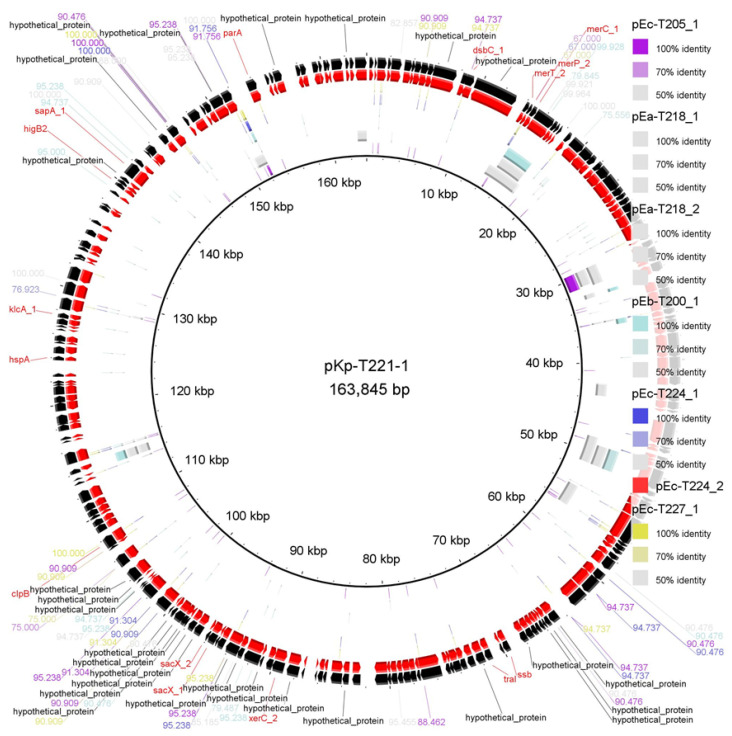
Comparative genome alignment of pKp-T221_1 with other reconstructed plasmid-like assemblies using BRIG. The central ring represents the reference assembly (pKp-T221_1), annotated with coding sequences (CDSs) from its GenBank files. The outer rings represent assemblies from *Enterobacterales* isolates (pEa-T218_1, pEa-T218_2, pEc-T205_1, pEc-T224_1, pEc-T224_2, pEc-T227_1, pEb-T200_1), where colour gradations indicate the degree of sequence identity (100%, 70%, 50%). Identity rings depict local sequence similarity and not circularity or synteny. BRIG (BLAST Ring Image Generator v0.95) [22] was used for visualisation, with black tick marks indicating predicted structural proteins or phage-related features. Assemblies are shown as circular representations for clarity, although physical circularity and structural integrity were not experimentally verified.

To explore sequence-level conservation among plasmid-like assemblies, the largest plasmid-derived contigs, pKp-T221_1 (163,845 bp) from *K. pneumoniae*, were used as a reference for comparative alignment in BRIG [22] (Figure 3).

The alignment revealed significant sequence heterogeneity between pKp-T221_1 and the other assemblies, based on local sequence alignments, indicating limited overall conservation among *Enterobacterales* assemblies (Figure 3). pEc-T205_1 (*E. coli*) displayed several large high-identity regions (~60–110 kbp and ~140–155 kbp), suggesting partially conserved genetic modules that may represent shared plasmid-associated regions. Other assemblies (e.g., pEa-T218_1, pEb-T200_1, pEc-T227_1) exhibited limited, fragmented identity (70–90%), possibly due to shared resistance islands or mobile genetic elements. The lowest similarity was found in pEc-T224_2 and pEa-T218_2, suggesting possible divergence among plasmid-associated contigs or the environmental acquisition of distinct gene modules.

## 3. Discussion

**Figure 4:**
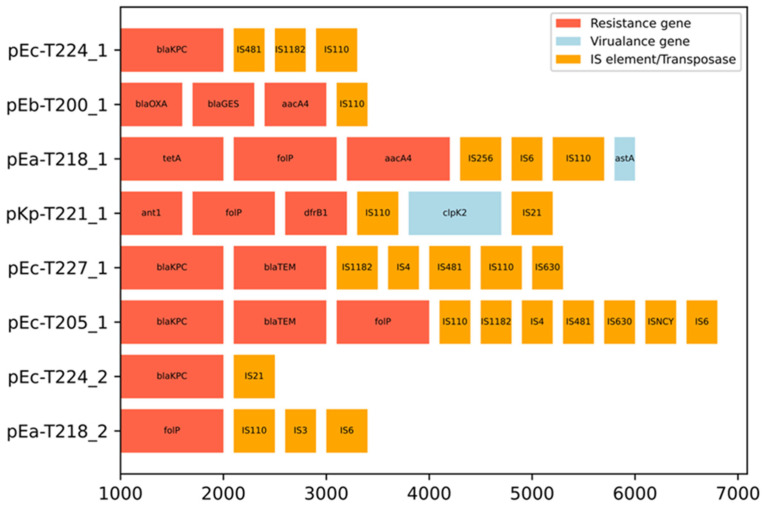
Gene neighbourhood diagram of resistance, virulence and mobile elements in eight *Enterobacteriaceae* plasmid-like assemblies. Resistance genes (red), virulence factors (light blue), insertion sequences/transposases (orange) are shown within ±5 kb regions. The annotations are based on CARD v4.0.2 [16], VFDB v6.0, VirulenceFinder v2.0.4 [23], ResFinder v4.4.2 [17], and ISEScan v1.7.2.3+galaxy0 [18]. The diagram illustrates the co-localisation of resistance genes with mobile elements and highlights the potential for horizontal gene transfer.

This study provides a functional and structural overview of eight putative plasmid-derived contig assemblies from marine *Enterobacterales* isolates collected in the central Adriatic Sea. The reconstructed plasmid-like sequences displayed different architectures and carried a number of ARGs, including *bla*_KPC_, *bla*_TEM_, *aacA4*, *tetA*, *folP*, *bla*_OXA_, *bla*_GES_, *dfrB1* and *ant1* (Table 1). These plasmid-like assemblies represent putative, fragmented plasmid reconstructions derived from MOB-suite clustering rather than complete circular plasmids. Assemblies pKp-T221_1 and pEc-T205_1 were particularly rich in resistance and showed regions of sequence similarity within the backbone and ARG-containing segments (Figure 2 and Figure 3). Gene neighbourhood analysis revealed that resistance genes were frequently located within or close to mobile regions flanked by IS elements and transposases (Figure 4). Most plasmid-like elements showed co-localisation of ARGs with mobile genetic elements (MGEs), including IS110, IS4, IS1182, IS6, and ISNCY, suggesting modular gene exchange and the potential for horizontal transmission.

For example, the *bla*_KPC_ loci in pEc-T205_1 and pEc-T224_1 showed conserved flanking regions with insertion sequences and hypothetical genes. While these arrangements suggest localised gene mobility and recombination-prone regions, they do not confirm full plasmid structural conservation. Such modularity is consistent with reports from wastewater clinical isolates [24]. The consistent co-localisation of ARGs with insertion sequences on all plasmid-like assemblies, regardless of host species (Figure 4), underlines the central role of MGEs in horizontal gene transfer [25,26]. However, functional transfer between strains was not experimentally demonstrated and remains a hypothesis requiring conjugation assays or long-read validation. The proximity of IS elements to genes such as *bla*_KPC_, *tetA* and *aacA4* suggests that these loci are potentially mobilisable across genetic contexts [2]. This architecture supports the concept of a marine mobilome, facilitating exchange of resistance determinants between different taxa [27].

Comparative BRIG mapping revealed pronounced sequence heterogeneity among plasmid-like assemblies (Figure 2 and Figure 3). Shared sequence blocks between *E. coli*, *E. bugandensis* and *K. pneumoniae* assemblies suggest common environmental resistance modules [28]. The large number of hypothetical proteins observed also highlights the prevalence of uncharacterised open reading frames typical of environmental metagenomes [29].

The presence of clinically important ARGs such as *bla*_KPC_, *bla*_GES_ and *aacA4* in marine *Enterobacterales* strongly suggests an anthropogenic influence, possibly related to coastal contamination by municipal or aquaculture effluents [4]. Several assemblies shared replicon signatures typical of IncA/C2 and IncP6 plasmids—backbones well-known from clinical carbapenemase vectors [30]. Here, these replicon types are inferred from sequence markers and not confirmed through complete circularisation. Their detection in multiple taxa supports the idea that environmental compartments may act as repositories for plasmid elements with clinical relevance. The detection of IncA/C2 and IncP6 replicon sequences indicates a potential broad host range [24]. IncA/C plasmids are known to replicate across multiple proteobacterial classes [24] while IncP 6 plasmids such as Rms149 have shown cross-genus replication in *Pseudomonas* and *E. coli* [31]. Although such replication potential cannot be directly confirmed here, the presence of these replicon motifs underscores environmental persistence and adaptability.

The BLAST-based comparisons revealed that plasmid-like assemblies share partial sequence identity with known clinical plasmids carrying blaKPC, blaNDM or blaIMP (e.g., p11A14057org1_B_NDM, PV023083.1; pLB_GSMA000, CP130614.1; pM12-KPC, CP093216.1). These similarities reflect the presence of conserved ARG modules and mobile elements rather than identical plasmid backbones. Thus, while sequence identity supports a shared gene pool between environmental and clinical sources, direct plasmid transfer cannot be inferred.

Virulence determinants were limited. The *astA* gene in pEa-T218_1 encodes the heat-stable EAST-1 enterotoxin, frequently found in both environmental and commensal *E. coli* [32,33]. Its presence here likely reflects environmental persistence rather than clinical virulence. Likewise, *clpK2* was annotated in pKp T221_1 but functions primarily as a heat-shock response gene, not a virulence factor.

Assemblies attributed to *K. pneumoniae* (pKp T221_1) and *E. coli* (pEc T205_1, pEc T224_1, pEc T227_1) contained ARGs linked to IncA/C2 and IncP6 replicons, consistent with previously described environmental and clinical hybrid plasmids but here represented by partial contig clusters. *E. coli* serves as a key environmental sentinel [34], and the co-occurrence of ARGs and MGEs in these isolates indicates plasmid exchange potential across species boundaries [35]. The *E. bugandensis* assembly pEb T200_1 carried *bla*_GES_, *bla*_OXA_ and *aacA4*, mirroring gene configurations described in hospital and aquaculture isolates [36]. *E. asburiae* assemblies (pEa T218_1, pEa T218_2) contained *folP* and *aacA4*, along with multiple IS elements, consistent with its recognised role as an opportunistic environmental reservoir [37].

### 3.1. Comparative Resistome Features in Adriatic Plasmid Assemblies

Compared with other Adriatic studies that used culture-independent metagenomics or culture-based profiling of resistance genes in wastewater, beach waters, or sediments [10,12,13], our study provided gene-level resolution of putative plasmid-derived contig assemblies, enabling insights into plasmid mobility potential and host range.

Replicon typing using PlasmidFinder identified IncA/C2 and IncP6 markers in several plasmid-like assemblies, which are also common in clinical and sewage-impacted environments. These replicon types, previously reported in studies near a submarine outfall in Split [38], support their persistence in both clinical and environmental settings. Similarly, MOB typing with MOB-suite classified most assemblies as MOBP or MOBQ, offering gene- and replicon-level resolution beyond the integron-based ARG co-localisation and IS-flanking pattern approaches used in previous Adriatic studies [9,13].

Insertion sequences (ISs) such as IS110, IS1182, and IS4 flanked multiple ARGs in our data, often in conserved arrangements previously reported in anthropogenically impacted zones [39]. This supports the existence of shared mobilisation pathways and conserved cassette architectures across distinct coastal niches.

The ARGs observed (e.g., *bla*_KPC_, *aacA4*, *tetA*) in our dataset also appear in other coastal surveys, often near tourism zones, ports, or hospital discharges [10,13], reinforcing their association with anthropogenic impact.

In summary, the comparison highlights that clinically relevant ARGs in Adriatic coastal environments are embedded in modular, mobilisable, plasmid-associated fragments. These findings support the One Health concept, linking environmental resistomes with human and animal antibiotic resistance networks. A comparative overview of ARGs, plasmid features, and environmental pressures in Adriatic studies is provided in Supplementary Table S1.

### 3.2. Methodological Strengths and Limitations

In this study, a reproducible pipeline was used to characterise putative plasmid-derived contig assemblies associated with ARGs from *Enterobacterales* in the environment. Carbapenem-resistant isolates were screened with selective media, followed by plasmid extraction, S1-PFGE profiling, Illumina-based NGS and de novo assembly with SPAdes [40]. Functional annotation and plasmid typing were performed using Prokka v1.14.6+galaxy1 [14], MOB-suite (v3.1.0+galaxy0) [15], PlasmidFinder (v2.1.1) [21], ISEScan (v1.7.2.3+galaxy0) [18], ResFinder (v4.4.2) [17], and CARD-RGI (v4.0.2) [16], with visualisation via EasyFig (v2.2.5) [19] and Proksee v1.0.0a6 (web application; https://proksee.ca, accessed on 30 June 2025) [41]. This integrated workflow enabled gene-level characterisation of resistance determinants and mobile elements across multiple taxa. However, due to the intrinsic limitations of short-read sequencing, the reconstructed sequences represent fragmented assemblies and are not complete, circular plasmids. The MOB-recon module groups contigs based on shared plasmid markers (e.g., relaxases, replicons), but does not confirm physical linkage between distant contigs, structural integrity, or plasmid circularity. As such, inferences regarding plasmid architecture, gene synteny, or mobility potential must be interpreted cautiously and considered hypotheses rather than confirmed biological entities.

### 3.3. Future Directions

Further studies should include functional validation of plasmid mobility through conjugation or transformation assays to assess the actual transferability of resistance elements. The use of long-read sequencing platforms such as Oxford Nanopore Technologies [42] or Pacific Biosciences [43] is highly recommended to reconstruct complete plasmid sequences, resolve repetitive elements, and confirm circularity. Combining long-read and short-read technologies (hybrid assembly) would provide a more accurate representation of plasmid structures.

Environmental monitoring should be extended to include marine sediments, biofilms, and wastewater-impacted coastal zones, which may harbour ARG reservoirs not detectable in seawater alone. Finally, longitudinal and seasonal studies are needed to track the dynamics of ARG-carrying plasmids over time and assess the impact of tourism, stormwater, and sewage influx on ARG dissemination in marine ecosystems.

## 4. Materials and Methods

### 4.1. Study Area and Seawater Sampling

Seawater samples were collected in September 2021 from the coastal area near the mouth of the Trstenik stream (43.501982° N, 16.465948° E) at a depth of approximately 1 m. Sampling was conducted from the shore using sterile 1-litre bottles and transported to the laboratory in a portable refrigerator maintained at approximately 4 °C. At the time of sampling, the seawater temperature was 24 °C.

The selected site, adjacent to the public Radoševac beach in the city of Split, is an area of significant anthropogenic influence, making it particularly suitable for assessing environmental antibiotic resistance dissemination. The Trstenik stream discharges directly into the marine environment and passes through a densely populated urban zone characterised by mixed land use, including residential and healthcare infrastructure. The area is subject to potential leakage from private septic tanks into the stormwater drainage network, especially during rainfall, contributing to non-point source pollution.

The proximity of the Clinical Hospital Center Split, a major tertiary care facility, further increases the likelihood of pharmaceutical and microbial contaminants entering the aquatic environment [9]. This location therefore serves as a relevant environmental interface between urban effluents, including potential hospital-derived discharges, and recreational coastal waters, representing a plausible conduit for the mobilisation and dissemination of antibiotic-resistant bacteria (ARB) and antibiotic resistance genes (ARGs) into the marine ecosystem. The site’s high human exposure potential, combined with its complex contamination profile, supports its selection as a critical surveillance point within a One Health framework.

### 4.2. Extraction of Plasmid DNA

Carbapenem-resistant *Enterobacteriaceae* (CRE) isolates were first cultured on MacConkey agar (Biolife Italiana S.r.l., Milan, Italy) supplemented with 2 µg/mL meropenem (MEM) (Sigma-Aldrich, St. Louis, MO, USA) and incubated overnight at 37 °C. Single colonies were then inoculated into 250 mL Luria–Bertani (LB) broth containing the same antibiotic concentration and grown under shaking conditions to achieve a high plasmid yield.

Plasmid DNA was extracted from the cultured cells using the PureYield™ Plasmid Midiprep System (Promega, Madison, WI, USA) according to the manufacturer’s instructions. Elution was performed using the Eluator™ Vacuum Elution Device (Promega, Madison, WI, USA), which allowed efficient recovery of plasmid DNA under vacuum pressure. The concentration and purity of the eluted plasmid DNA was determined spectrophotometrically using a NanoPhotometer^®^ N60 (Implen GmbH, Munich, Germany).

S1-nuclease pulsed-field gel electrophoresis (S1-PFGE) was performed to detect specific resistance-associated plasmids. After electrophoresis, 24 different plasmid bands were excised from the agarose gels. These bands were selected based on a combination of factors, including Southern blot hybridisation results with a digoxigenin-labelled *bla*_KPC_ gene fragment probe, antimicrobial susceptibility profiles (antibiograms), species identification and the origin of the isolate.

Plasmid DNA from the excised bands was extracted using the QIAEX^®^ II Gel Extraction Kit (QIAGEN GmbH, Hilden, Germany) according to the manufacturer’s protocol to obtain purified plasmid DNA suitable for next-generation sequencing (NGS) library preparation and downstream analyses.

### 4.3. Preparation of Plasmid Libraries and Illumina Sequencing

Plasmid DNA libraries were prepared using the Illumina^®^ DNA Prep Kit (Cat. No. 20060060, Illumina Inc., San Diego, CA, USA), which uses bead-linked transposomes (BLTs) for simultaneous fragmentation and labelling of DNA with adapter sequences in a single enzymatic reaction. The library preparation workflow was performed according to the manufacturer’s protocol, with adjustments made for multiplexing 24 samples.

On-bead tagmentation was performed using the bead-linked transposome method. In this step, 24 plasmid DNA samples were simultaneously fragmented and tagged with Illumina adapters by the action of BLTs. This step allowed the integration of sequencing adapters while minimising DNA input requirements.

After tagmentation, a post-tagment cleanup was performed directly on the BLTs to remove reagent residues and wash the adapter-ligated DNA to ensure optimal conditions for subsequent amplification.

A limited-cycle PCR amplification step was performed to enrich the tagmented fragments and complete the addition of full-length adapter sequences. This reaction incorporated the index 1 (i7) and index 2 (i5) adapters as well as the flow cell binding sequences required for clustering during sequencing.

Sample multiplexing was enabled by the use of Nextera™ DNA CD Indexes (Cat. No. 20018707, Illumina), which allow parallel processing and demultiplexing of up to 24 plasmid libraries in a single sequencing run.

Amplified libraries were subjected to double-sided bead purification using the Illumina Purification Beads (IPB), which are part of the DNA Prep workflow. This step selectively removed adapter dimers and small DNA fragments, resulting in clean libraries suitable for sequencing.

After purification, the individual libraries were quantified using the Qubit™ dsDNA High Sensitivity Assay Kit (Life Technologies, Eugene, OR, USA) on a Qubit™ fluorometer and then normalised and pooled into a composite library to improve sequencing throughput and cost efficiency.

Sequencing was performed in-house on the Illumina iSeq™ 100 System using the iSeq™ 100 i1 Reagent v2 Kit (Cat. No. 20031371), which supports up to 322 sequencing cycles. This kit contains the iSeq™ 100 i1 Reagent Cartridge v2 for paired-end sequencing chemistry and the iSeq™ 100 i1 Flow Cell. Paired-end sequencing was performed with a read length of 2 × 151 bp.

### 4.4. Assembly and Annotation of Putative Plasmid-Derived Contigs

Raw paired-end reads from Illumina sequencing were subjected to initial quality control using FastQC (v0.11.9) [44] to assess sequence quality metrics. Adapter sequences and low-quality bases were removed using Trimmomatic (v0.39) [45], and the resulting reads were reassessed with FastQC [44] to confirm quality after trimming. High-quality reads were then assembled de novo using SPAdes (v3.14.1) [40] with the plasmid flag, which improves recovery of extrachromosomal elements from short-read data.

Assembly quality was assessed using QUAST (v5.2.0) [46], which provides metrics such as N50, total length, and GC content. Functional annotation of assembled contigs was performed using Prokka v1.14.6+galaxy1 [14], which identifies coding sequences (CDS), rRNAs, tRNAs, and other genomic features. Insertion sequences (ISs) were identified with ISEScan v1.7.2.3+galaxy0 [18], which uses HMM-based detection of known IS families.

Plasmid classification and typing were performed using the MOB-suite v3.1.0+galaxy0 [15], including Mob-recon for putative plasmid reconstruction, MOB typing (relaxase gene-based), and prediction of mobility classes. Incompatibility (Inc) typing was carried out using PlasmidFinder (v2.1.1) [21] and its curated replicon reference database. Assembly and annotation reports, including log files from the above tools, were merged using MultiQC (v1.11) to optimise verification and quality control [47].

To annotate antimicrobial resistance genes (ARGs) and virulence determinants, the plasmid contigs were screened using ABRicate v1.0.1+galaxy0 with the CARD v4.0.2, NCBI AMRFinderPlus and VFDB v6.0 [16], and cross-validated with ResFinder (v4.4.2) [17] and VirulenceFinder (v2.0.4) [23]. Only high-confidence hits with ≥90% identity and ≥60% coverage were retained for downstream analyses.

### 4.5. Putative Plasmid Sequence Clustering and Visualisation

Putative plasmid-derived assemblies were reconstructed from assembled genomic contigs using MOB-suite v3.0.0 [15], a toolset developed for plasmid identification and classification from short-read data. Specifically, the Mob-recon module [15] was used to extract and cluster contigs predicted to originate from plasmids based on replicon markers, relaxase genes, and mobilisation features. The resulting plasmid-like assemblies were assigned to primary clusters (e.g., AA002, AA364) based on sequence similarity and replicon typing (Table 3). It should be noted that these assemblies do not represent complete, circular plasmids but rather reconstructed genomic regions likely of plasmid origin.

To facilitate gene annotation and visualisation, contigs identified as plasmid sequences were annotated using Prokka v1.14.6+galaxy1 [14] which generated .gff files with predicted coding sequences, resistance genes, and mobile genetic elements. Insertion sequences (IS) were annotated using ISEScan [18], and the results were integrated into the final annotation files. All annotated .gff and .fna files were then converted to GenBank format (.gbk) using the “FASTA + GFF3 to GenBank/EMBL” converter [48] available on the Galaxy Europe platform (usegalaxy.eu).

Reconstructed plasmid-like assembly maps were generated using Proksee v1.0.0a6 (web application; https://proksee.ca, accessed on 30 June 2025) [41] (Supplementary Figures S1–S8) and EasyFig v2.2.5 [19] (Figure 1). These tools enabled the visualisation of gene positions, strand direction, insertion sequences, and other functional features. Comparative genomic analysis of the reconstructed assemblies was performed using the BLAST Ring Image Generator (BRIG v0.95) [22]. For this comparison, the largest reconstructed plasmid-like assembly, pKp-T221_1 (from *K. pneumoniae*, cluster AA860), was used as the reference genome. All other assemblies were aligned to this reference genome to assess structural similarity, shared ARG regions, and ARG synteny.

## 5. Conclusions

In this study, we characterised putative plasmid-derived contig assemblies from eight carbapenem-resistant *Enterobacterales* isolates recovered from the coastal waters of the central Adriatic Sea. These reconstructed assemblies contained clinically relevant antimicrobial resistance genes (ARGs)—including *bla*_KPC_, *bla*_TEM_, *aacA4*, *folP*, and *tetA*—together with multiple insertion sequences (e.g., IS110, IS6, IS1182) and replicon types indicative of potential mobility.

Although short-read data preclude definitive conclusions about plasmid circularity or complete architecture, the co-localisation of ARGs with IS elements and MOB markers across diverse bacterial taxa (e.g., *E. coli*, *K. pneumoniae*, *E. bugandensis*, *E. asburiae*) highlights the possible modularity and transfer potential of these resistance elements in the marine environment.

Comparative analysis with other Adriatic studies revealed recurring ARGs and mobilisation patterns across anthropogenically influenced coastal sites, including shared resistance genes (*bla*_KPC_, *aacA4*), IS elements, and replicon markers (IncA/C2, IncP6) characteristic of broad-host-range plasmids. These observations support the hypothesis that coastal waters serve as reservoirs for plasmid-associated ARGs, shaped by wastewater, urban runoff, and maritime activity.

This work underscores the value of integrative, genome-based environmental surveillance to monitor ARG dissemination in marine ecosystems under the One Health framework. Future efforts incorporating long-read sequencing and functional validation are essential to resolve plasmid structure and confirm transferability, enabling more precise risk assessments of resistance transmission across ecological boundaries.

## Figures and Tables

**Table 1:** Functional overview of antibiotic resistance genes (ARGs) identified in marine *Enterobacterales* plasmid-like assemblies *.

Resistance Class	Gene	Mechanism of Action	Target Antibiotic(s)	Plasmid(s) Detected	Host Species
β-lactamase	*bla*_KPC_	Class A serine carbapenemase (hydrolyzes β-lactams)	Carbapenems, penicillins, cephalosporins	pEc-T224_1, pEc-T224_2, pEc-T227_1, pEc-T205_1	*E. coli*
β-lactamase	*bla*_TEM_	Broad-spectrum β-lactamase	Penicillins, early cephalosporins	pEc-T227_1, pEc-T205_1	*E. coli*
β-lactamase	*bla*_GES_	ESBL; some variants hydrolyze carbapenems	Cephalosporins, carbapenems (variant-dependent)	pEb-T200_1	*E. bugandensis*
β-lactamase	*bla*_OXA_	Class D β-lactamase (oxacillinase)	Penicillins, cephalosporins	pEb-T200_1	*E. bugandensis*
Aminoglycoside	*aacA4*	Aminoglycoside acetyltransferase (modifies 6′-amine group)	Gentamicin, tobramycin, netilmicin	pEa-T218_1, pEb-T200_1	*E. asburiae*, *E. bugandensis*
Aminoglycoside	*ant(3″)-Ia (ant1)*	Streptomycin adenylyltransferase	Streptomycin	pKp-T221_1	*K. pneumoniae*
Sulfonamide	*folP*	Dihydropteroate synthase variant (target alteration)	Sulfonamides	pEa-T218_1, pEa-T218_2, pKp-T221_1, pEc-T205_1	*E. asburiae*, *K. pneumoniae*, *E. coli*
Trimethoprim	*dfrB1*	Trimethoprim-resistant dihydrofolate reductase	Trimethoprim	pKp-T221_1	*K. pneumoniae*
Tetracycline	*tetA*	Efflux pump (major facilitator superfamily)	Tetracyclines	pEa-T218_1	*E. asburiae*

* Retrieved from the CARD database [20].

**Table 2:** Functional annotation of reconstructed plasmid-like assemblies from marine *Enterobacterales*: ARG content, incompatibility groups, insertion sequences, virulence determinants, and MOB types.

Plasmid	Resistance Genes (CARD/NCBI/ResFinder)	Incompatibility Groups (PlasmidFinder)	Insertion Sequences (ISEScan)	Virulence Genes (VFDB/VirulenceFinder)	MOB Types
pEa-T218_1	*tetA; folP; aacA4*		IS256; IS6; IS110; IS91	*astA*	
pEa-T218_2	*folP*;		IS110; IS3; IS6		
pKp-T221_1	*ant1*; *folP*; *dfrB1*	IncA/C2	IS21; IS4; IS5; ISNCY; IS110; ISL3; IS91	*clpK2*	MOBP
pEc-T224_1	*bla*_KPC_	IncP6	IS481; IS1182; IS110		MOBP
pEc-T224_2	*bla*_KPC_		IS21		
pEc-T227_1	*bla*_KPC_; *bla*_TEM_	IncP6	IS1182; IS4; IS481; IS110; IS630		MOBP
pEb-T200_1	*bla*_OXA_; *bla*_GES_; *aacA4*		IS110		MOBQ
pEc-T205_1	*bla*_KPC_; *bla*_TEM_; *folP*	IncP6	IS110; IS1182; IS4; IS481; IS630; ISNCY; IS6		MOBP

Blank entries indicate features not detected or not assigned.

**Table 3:** Summary of putative plasmid-derived assemblies from MOB suite Mob-recon.

Plasmid-Derived Assembly	Species	Primary Cluster ID	Size (bp)	Contigs
pEa-T218_1	*E. asburiae*	AA002	77,664	33
pEa-T218_2	*E. asburiae*	AC303	20,793	6
pKp-T221_1	*K. pneumoniae*	AA860	163,845	85
pEc-T224_1	*E. coli*	AA364	28,914	11
pEc-T224_2	*E. coli*	AA364	7544	6
pEc-T227_1	*E. coli*	AA364	35,093	10
pEb-T200_1	*E. bugandensis*	AC303	24,221	8
pEc-T205_1	*E. coli*	AA364	43,424	3

## Data Availability

The Illumina raw sequence data generated in this study have been deposited in the European Nucleotide Archive (ENA) under project accession number PRJEB49384. The individual run accession numbers are as follows: pEa-T218_1—ERX15131161; pEa-T218_2—ERX15131162; pEb-T200_1—ERX15131163; pEc-T205_1—ERX15131164; pEc-T224_1—ERX15131165; pEc-T224_2—ERX15131166; pEc-T227_1—ERX15131167; pKp-T221_1—ERX15131168. The FASTA sequences of the eight reconstructed plasmid-like assemblies in this study have been deposited in the NCBI GenBank database under accession numbers PV956236–PV956243 and will be made publicly available after their release.

## Supplementary Materials

The following supporting information can be downloaded at: https://www.mdpi.com/article/10.3390/ijms262210910/s1.

## Abbreviations

The following abbreviations are used in this manuscript:

ARGs	antibiotic resistance genes
IS	insertion sequences
Inc	incompatibility
MGEs	mobile genetic elements
CRE	carbapenem-resistant *Enterobacteriaceae*
MEM	meropenem
LB	Luria–Bertani
S1-PFGE	S1-nuclease pulsed-field gel electrophoresis
NGS	next generation sequencing
BLTs	bead-linked transposomes
IPB	Illumina Purification Beads
