# Supplementary material for: Characterisation of Plasmid-Associated Antimicrobial Resistance Genes in Coastal Marine Enterobacterales from the Central Adriatic Sea: De Novo Assembly and Bioinformatic Profiling
Source: Int J Mol Sci. 2025 Nov 11;26(22):10910. doi: 10.3390/ijms262210910 (PMC12652098; doi:10.3390/ijms262210910)
Supplement: Supplementary file 1 [file ijms-26-10910-s001.zip › ijms-3891215-final supplementary.pdf]

Supporting information for:

Characterisation of Plasmid-Associated Antimicrobial Resistance Genes in Coastal Marine *Enterobacterales* from the Central Adriatic Sea: De Novo Assembly and Bioinformatic Profiling

Ivica Šamanić [1,*], Mia Dželalija [1], Ema Bellulovich [2], Hrvoje Kalinić [3], Slaven Jozić [4], Marin Ordulj [5], Nikolina Udiković-Kolić [6] and Ana Maravić [1,*]

[1] Department of Biology, Faculty of Science, University of Split, 21000 Split, Croatia; mdzelalij@pmfst.hr
[2] Center for Proteomics, Faculty of Medicine, University of Rijeka, 51000 Rijeka, Croatia; ema.bellulovich@medri.uniri.hr
[3] Department of Informatics, Faculty of Science, University of Split, 21000 Split, Croatia; hrvoje.kalinic@pmfst.hr
[4] Laboratory of Marine Microbiology, Institute of Oceanography and Fisheries, 21000 Split, Croatia; sjozic@izor.hr
[5] University Department of Marine Studies, University of Split, 21000 Split, Croatia; mordulj@unist.hr
[6] Division for Marine and Environmental Research, Ruđer Bošković Institute, 10000 Zagreb, Croatia; nudikov@irb.hr
* Correspondence: isamanic@pmfst.hr (I.Š.); amaravic@pmfst.hr (A.M.)

Supplementary Figures S1–S8. Annotated maps of putative plasmid-derived contig assemblies from marine *Enterobacterales*.

Circular genome visualisations of eight putative plasmid-derived contig assemblies reconstructed from short-read de novo assemblies of marine *Enterobacterales* isolates: pEa-T218_1, pEa-T218_2, pKp-T221_1, pEc-T224_1, pEc-T224_2, pEc-T227_1, pEb-T200_1, and pEc-T205_1. Assemblies were annotated and visualised using the Proksee (v1.0.0a6) platform (Grant et al., 2023), which integrates Prokka for gene prediction, CARD-RGI (Resistance Gene Identifier) for antimicrobial resistance gene (ARG) detection, mobileOG-db for mobile genetic element (MGE) identification, and a GC content track for local sequence composition. These contig assemblies, clustered by MOB-suite based on plasmid-associated features, are presented as circularised visualisations, although physical circularity and structural integrity were not experimentally confirmed. The maps support the functional annotations discussed in the main text and provide graphical insight into ARG and MGE distribution.

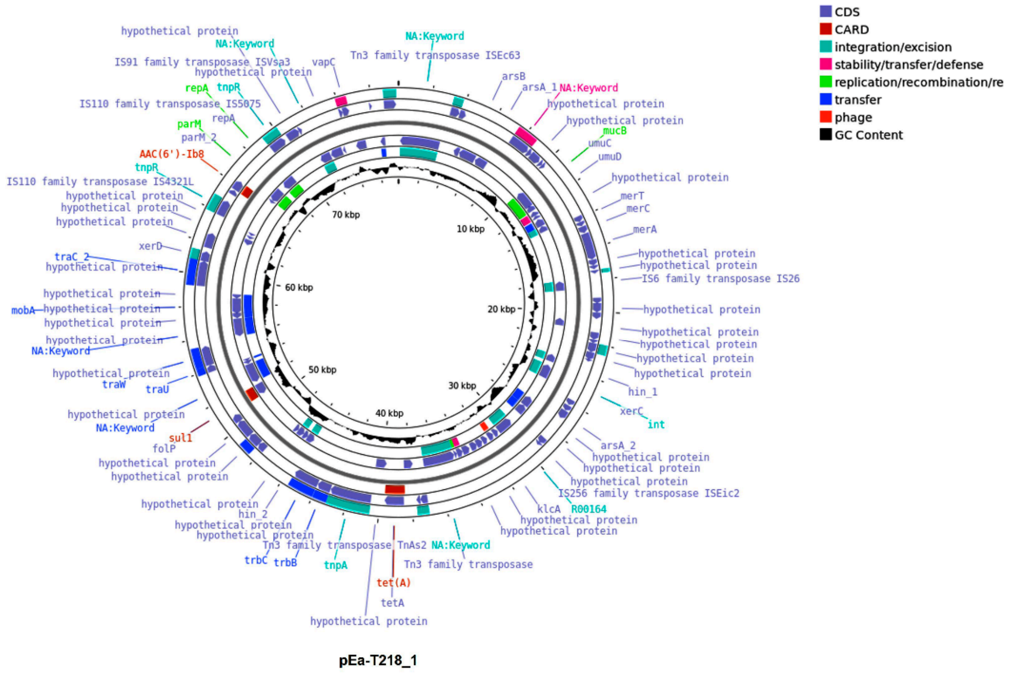

Supplementary Figure S1_ Annotated Map of the Putative Plasmid-Derived Contig Assembly pEa-T218_1_phs

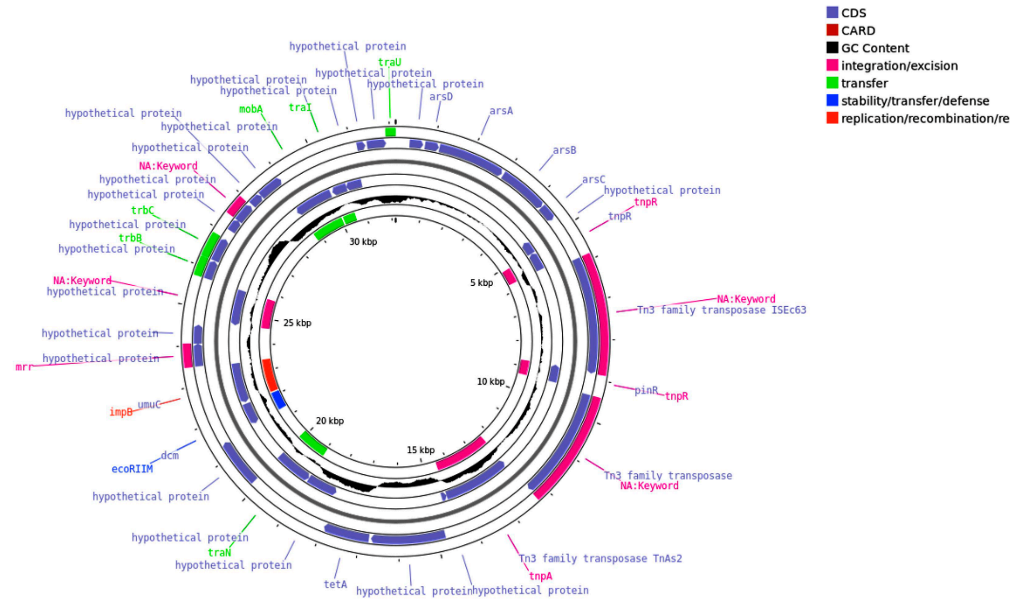

pEa-T218_2

Supplementary Figure S2_Annotated Map of the Putative Plasmid-Derived Contig Assembly pEa-T218_2_phs

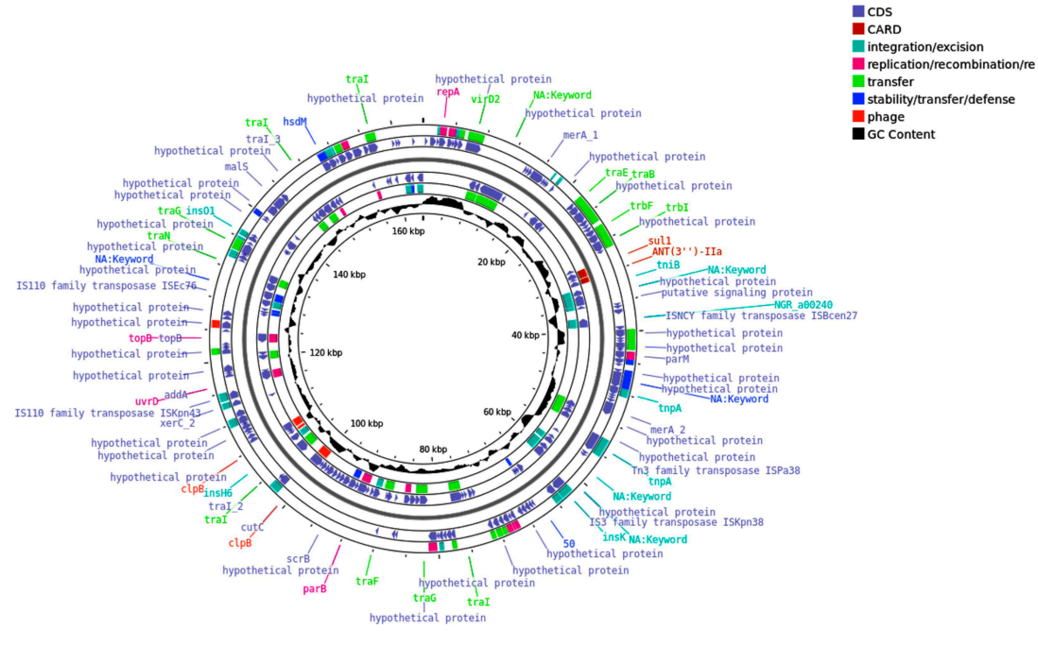

pKp-T221_1

Supplementary Figure S3_Annotated Map of the Putative Plasmid-Derived Contig Assembly pKp T221_1_phs

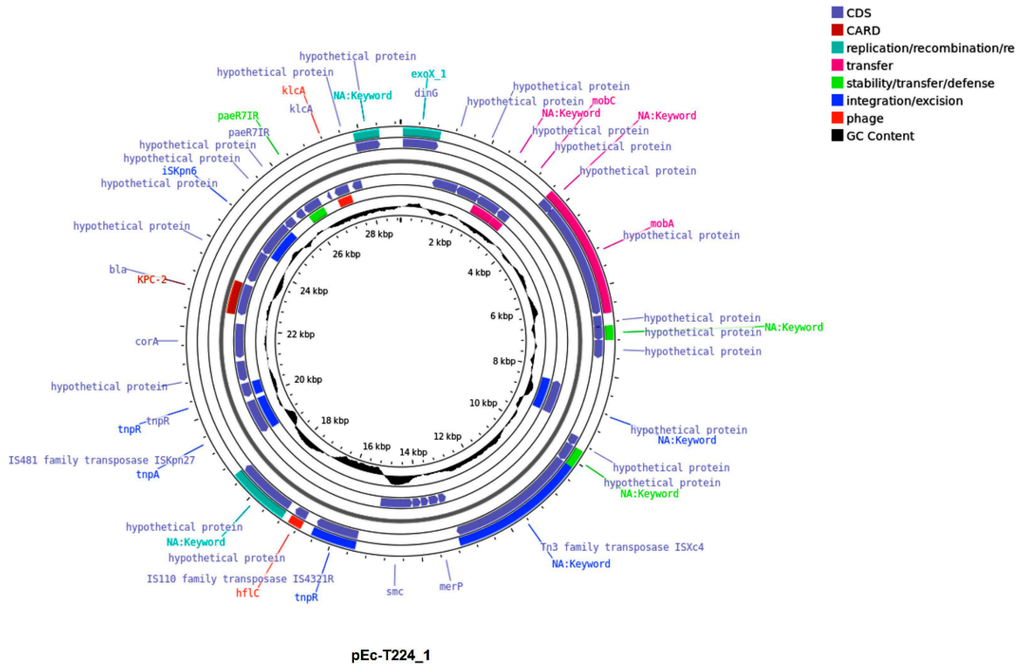

Supplementary Figure S4_Annotated Map of the Putative Plasmid-Derived Contig Assembly pEc-T224_1

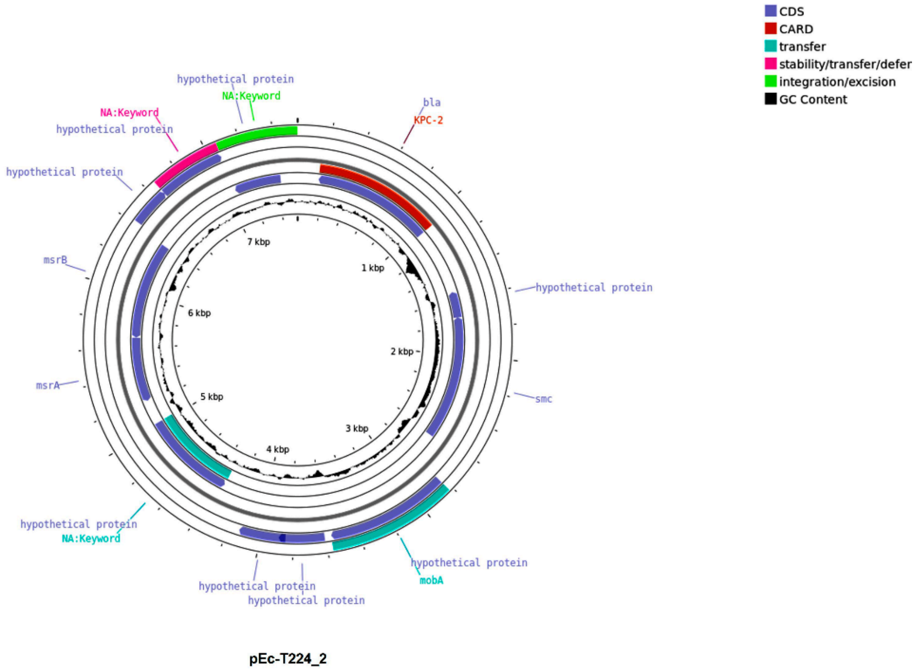

Supplementary Figure S5_Annotated Map of the Putative Plasmid-Derived Contig Assembly_pEc-T224_2

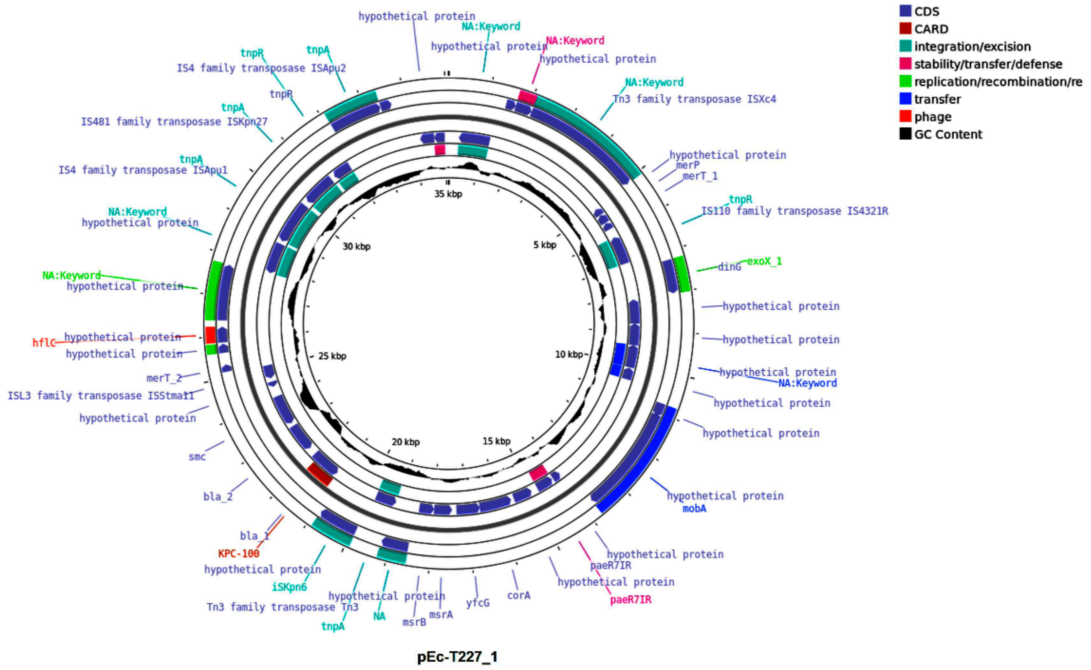

Supplementary Figure S6_Annotated Map of the Putative Plasmid-Derived Contig Assembly_pEc-T227_1

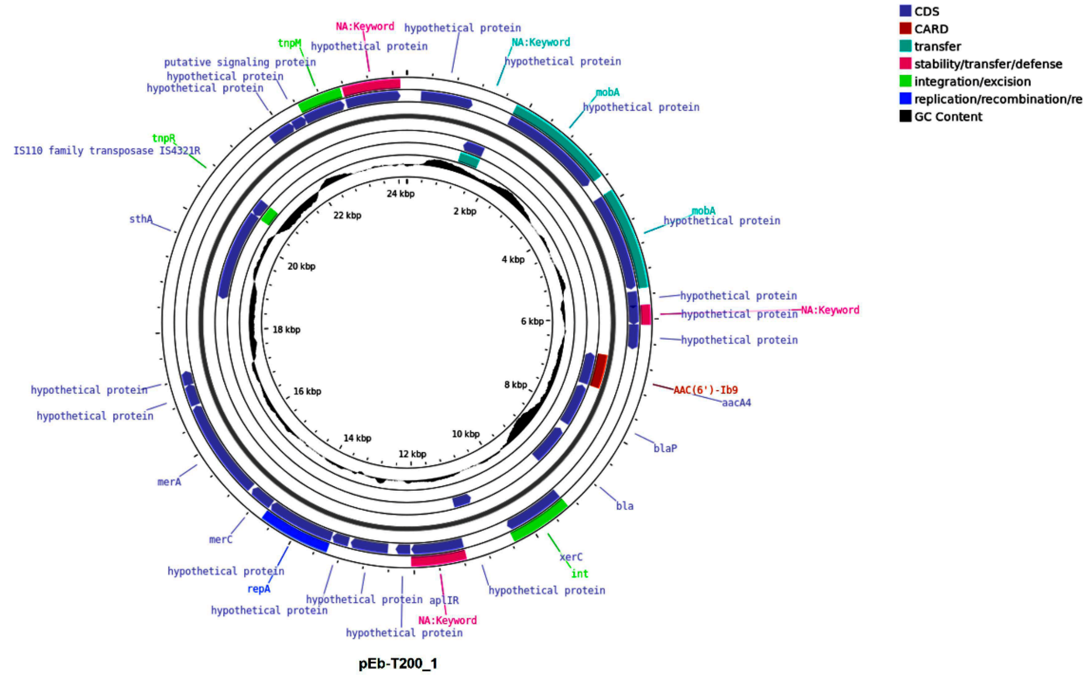

Supplementary Figure S7_Annotated Map of the Putative Plasmid-Derived Contig Assembly_pEb-T200_1

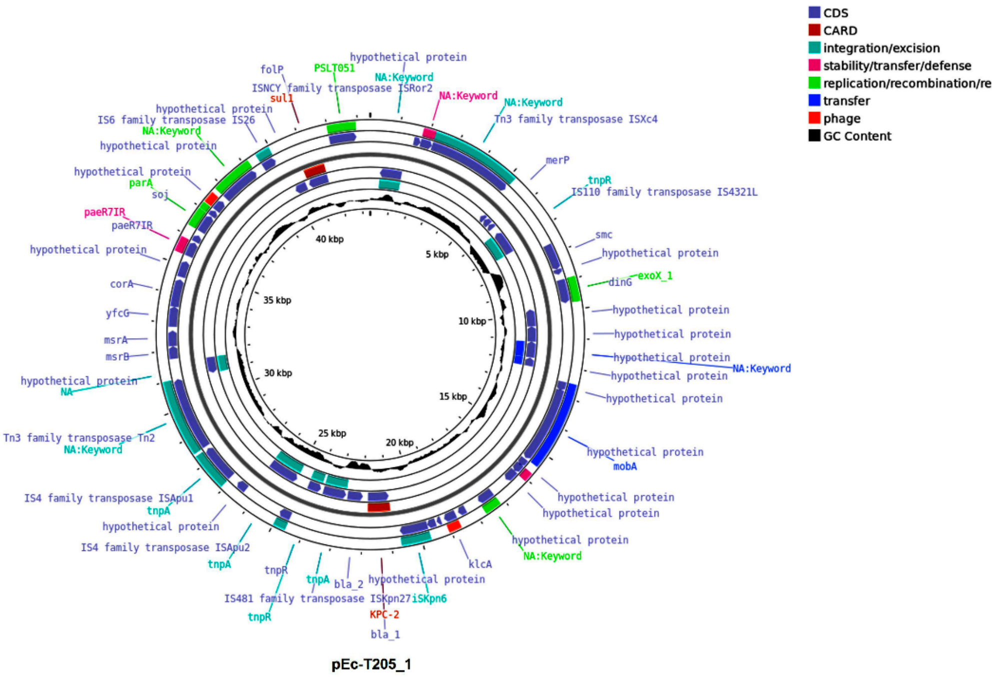

Supplementary Figure S8_ Annotated Map of the Putative Plasmid-Derived Contig Assembly_pEc-T205_1

References

Grant, J.R., Enns, E., Marinier, E., Mandal, A., Herman, E.K., Chen, C.Y., Graham, M., Van Domselaar, G. and Stothard, P. (2023) 'Proksee: In-depth characterization and visualization of bacterial genomes', *Nucleic Acids Research*, 51(W1), pp. W484–W492. Available at: https://doi.org/10.1093/nar/gkad326.

Supplementary Table S1. Comparative overview of ARGs, inferred plasmid features, and anthropogenic pressures across Adriatic coastal studies. Includes data from this study and six published datasets, highlighting gene content, mobility markers, and environmental contexts. Plasmid features are inferred from sequence data and do not confirm complete plasmid structures.

**Study Focus**	**ARGs Detected**	**Plasmid Replicons**	**MOB Types**	**IS Elements**	**Transferability Evidence**	**Host Species**	**Sample Sites**	**Anthropogenic Pressures**	**References**
**Plasmid-Associated ARGs in Marine *Enterobacterales* from the Central Adriatic**	*bla*$_{KPC}$, *bla*$_{TEM}$, *bla*$_{GES}$, *bla*$_{OXA}$, *aac*$_{A4}$, *ant1*, *folP*, *dfrB1*, *tetA*	IncA/C2, IncP6	MOBP, MOBQ	IS110, IS4, IS6, IS1182, IS630	MOB typing; *tra* genes not confirmed	*E. coli*, *K. pneumoniae*, *E. bugandensis*, *E. asburiae*	Radoševac (Split)	Hospital discharge, sewage	This Study
**Carbapenemase-Producing *Enterobacterales* from a Submarine Outfall near Split**	*bla*$_{KPC-2}$, *bla*$_{OXA-48}$, *bla*$_{TEM}$, *sul1*, *qnrS*, *aadA*, *aac(6')-Ib*	IncA/C2, IncL, IncFII	MOBP	IS26, ISKpn19	Conjugation confirmed	*E. coli*, *K. pneumoniae*, *C. freundii*	Submarine outfall near Split	Hospital discharge	Kvesić et al., 2022
**Antibiotic Resistance Gradients in Coastal Waters of the Adriatic**	*bla*$_{KPC}$, *qnr*S, *tetA*, *sul1*, *ermB*	IncFII	MOBP	ISKpn6, ISKpn7	Conjugation inferred	*E. coli*, *Klebsiella* spp.	Multiple marine and coastal points	Tourism, sewage	Kvesić et al., 2021
**Colistin-Resistant Bacteria and *mcr*-1 Detection in Coastal Waters**	*tetA*, *sul1*, *qnrS*, *ermB*, *bla*$_{CTX-M}$	IncP1, IncQ (implied)	MOBQ, MOBF (implied)	IS26, ISEcp1 (reported by gene context)	Plasmid markers present	*E. coli*, *Klebsiella*, *Serratia* spp.	Tourist beaches, aquaculture	Aquaculture, tourism	Šamanić et al., 2021)

**Vancomycin-Resistant *Enterococcus* in Coastal Waters**	*vanA*, *vanB*, *aac(6′)-Ie*, *aph(2″)-Ia*, *aph(2″)-Ib/Id*	Not applicable	Not applicable	Not reported	Not applicable	*E. faecium*	Wastewater, beach, clinical	Hospital, urban wastewater	Dželalija et al., 2023
**Biogeographical Shifts in ARGs along an Adriatic Trophic Gradient**	*sul1*, *qnrS*, bla_{OXA}, *intI1*, *tetA*, bla_{VIM}, bla_{TEM}, *ermB*, *mphA*	Not specified	Not specified	ISs not detailed	Inferred via ARG-IS associations	Mixed marine bacteria	Central Adriatic gradient	Sewage, urban inputs	Dželalija et al., 2024
**Marine Resistome Dynamics across an Adriatic Trophic Gradient**	*sul1*, *qnrS*, bla_{OXA}, bla_{TEM}, *intI1*, macrolide, *van*, imipenem	Not specified	Not specified	ISs not detailed	Inferred via ARG-IS associations	Mixed marine bacteria	Broad Adriatic gradient	Sewage, ports, trophic gradient	Dželalija et al., 2023

References

1. Kvesic, M.; Samanic, I.; Novak, A.; Fredotovic, Ä.; Dzelalija, M.; Kamenjarin, J.; Barisic, I.; Tonkic, M.; Maravic, A. Submarine Outfalls of Treated Wastewater Effluents are Sources of Extensively- and Multidrug-Resistant KPC- and OXA-48-Producing Enterobacteriaceae in Coastal Marine Environment. *Frontiers in Microbiology* **2022**, 13, doi:10.3389/fmicb.2022.858821.

2. Kvesic, M.; Kalinic, H.; Dzelalija, M.; Samanic, I.; Andricevic, R.; Maravic, A. Microbiome and antibiotic resistance profiling in submarine effluent-receiving coastal waters in Croatia. *Environmental Pollution* **2022**, *292*, doi:10.1016/j.envpol.2021.118282.

3. Samanic, I.; Kalinic, H.; Fredotovic, Z.; Dzelalija, M.; Bungur, A.; Maravic, A. Bacteria tolerant to colistin in coastal marine environment: Detection, microbiome diversity and antibiotic resistance genes' repertoire. *Chemosphere* **2021**, *281*, doi:10.1016/j.chemosphere.2021.130945.

4. Dzelalija, M.; Kvesic, M.; Novak, A.; Fredotovic, Ä.; Kalinic, H.; Samanic, I.; Ordulj, M.; Jozic, S.; Barisic, I.; Tonkic, M.; et al. Microbiome profiling and characterization of virulent and vancomycin- resistant *Enterococcus faecium* from treated and untreated wastewater, beach water and clinical sources. *Science of the Total Environment* **2023**, *858*, doi:10.1016/j.scitotenv.2022.159720.

5. Dzelalija, M.; Fredotovic, Z.; Udikovic-Kolic, N.; Kalinic, H.; Jozic, S.; Samanic, I.; Ordulj, M.; Maravic, A. Large-Scale Biogeographical Shifts of Abundance of Antibiotic Resistance Genes and Marine Bacterial Communities as Their Carriers along a Trophic Gradient. *International Journal of Molecular Sciences* **2024**, *25*, doi:10.3390/ijms25010654.

6. Dzelalija, M.; Kvesic-Ivankovic, M.; Jozic, S.; Ordulj, M.; Kalinic, H.; Pavlinovic, A.; Samanic, I.; Maravic, A. Marine resistome of a temperate zone: Distribution, diversity, and driving factors across the trophic gradient. *Water Research* **2023**, *246*, doi:10.1016/j.watres.2023.120688.
