# Supplementary figures and images for: Characterisation of Plasmid-Associated Antimicrobial Resistance Genes in Coastal Marine Enterobacterales from the Central Adriatic Sea: De Novo Assembly and Bioinformatic Profiling
Source: Int J Mol Sci. 2025 Nov 11;26(22):10910. doi: 10.3390/ijms262210910 (PMC12652098; doi:10.3390/ijms262210910)

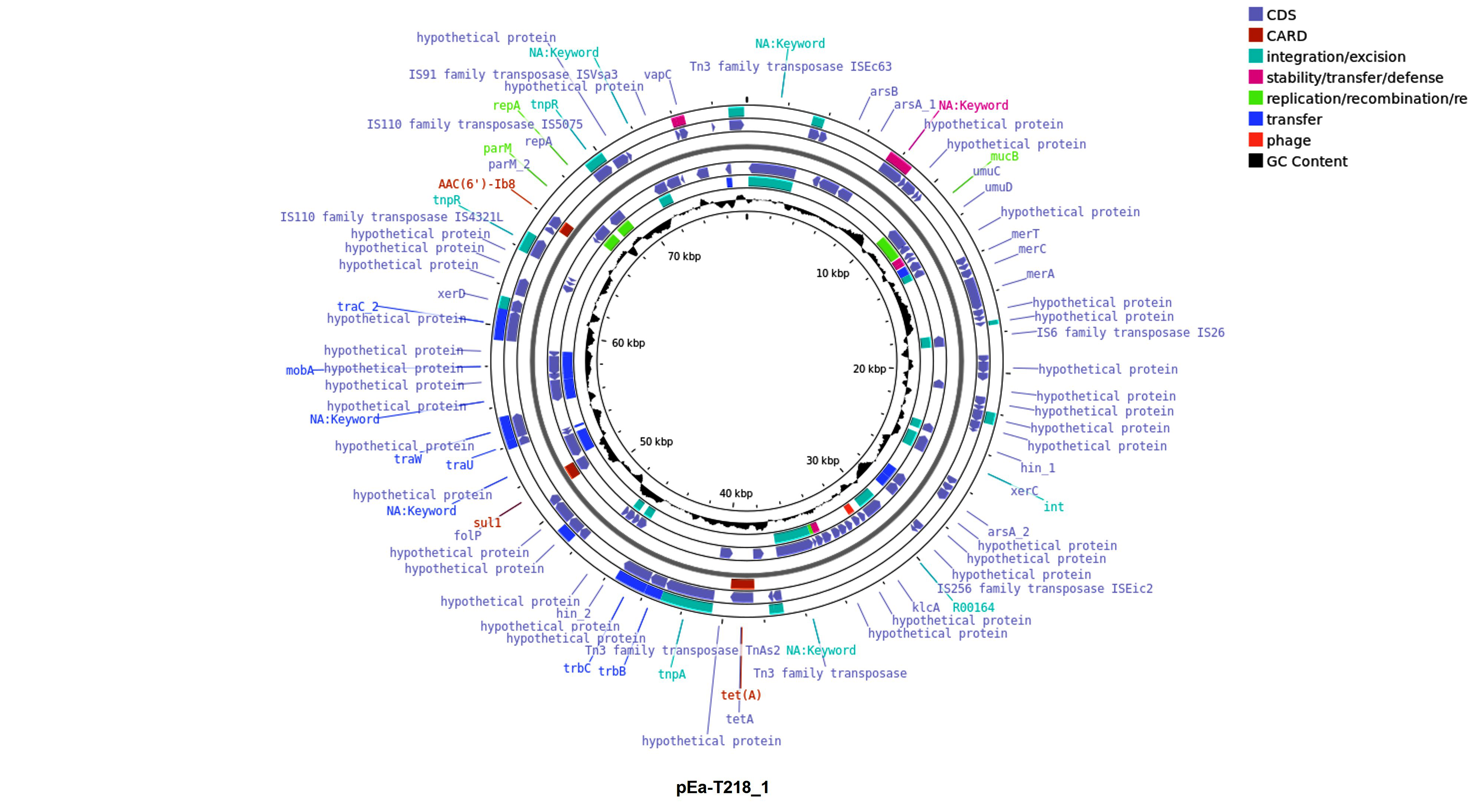

Supplement: Supplementary file 1 [file ijms-26-10910-s001.zip › Suplementary Figure_IJMS-3891215/Supplementary Figure S1_Annotated Map of the Putative Plasmid-Derived Contig Assembly pEa-T218_1_phs.tif]

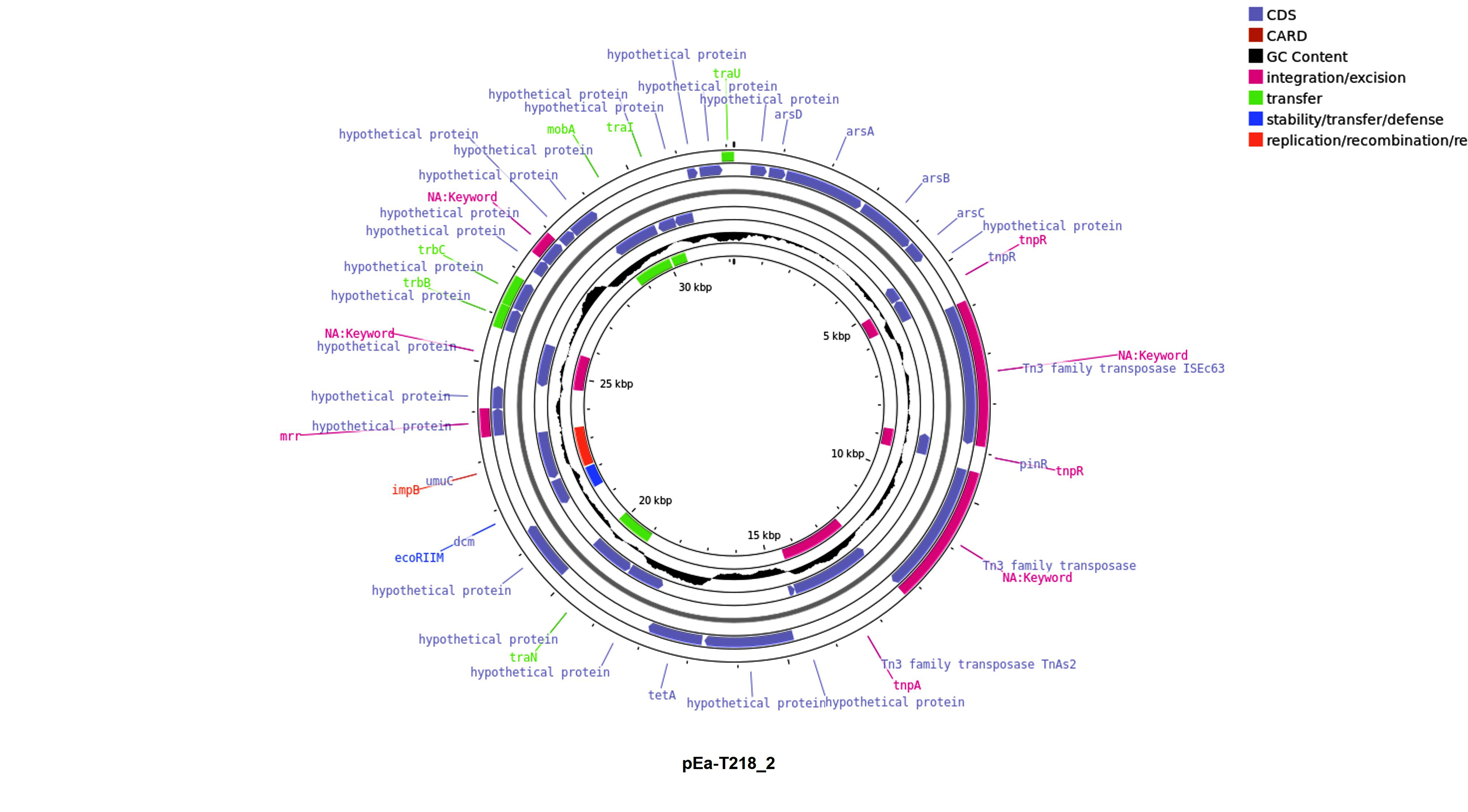

Supplement: Supplementary file 1 [file ijms-26-10910-s001.zip › Suplementary Figure_IJMS-3891215/Supplementary Figure S2_Annotated Map of the Putative Plasmid-Derived Contig Assembly pEa-T218_2_phs.tif]

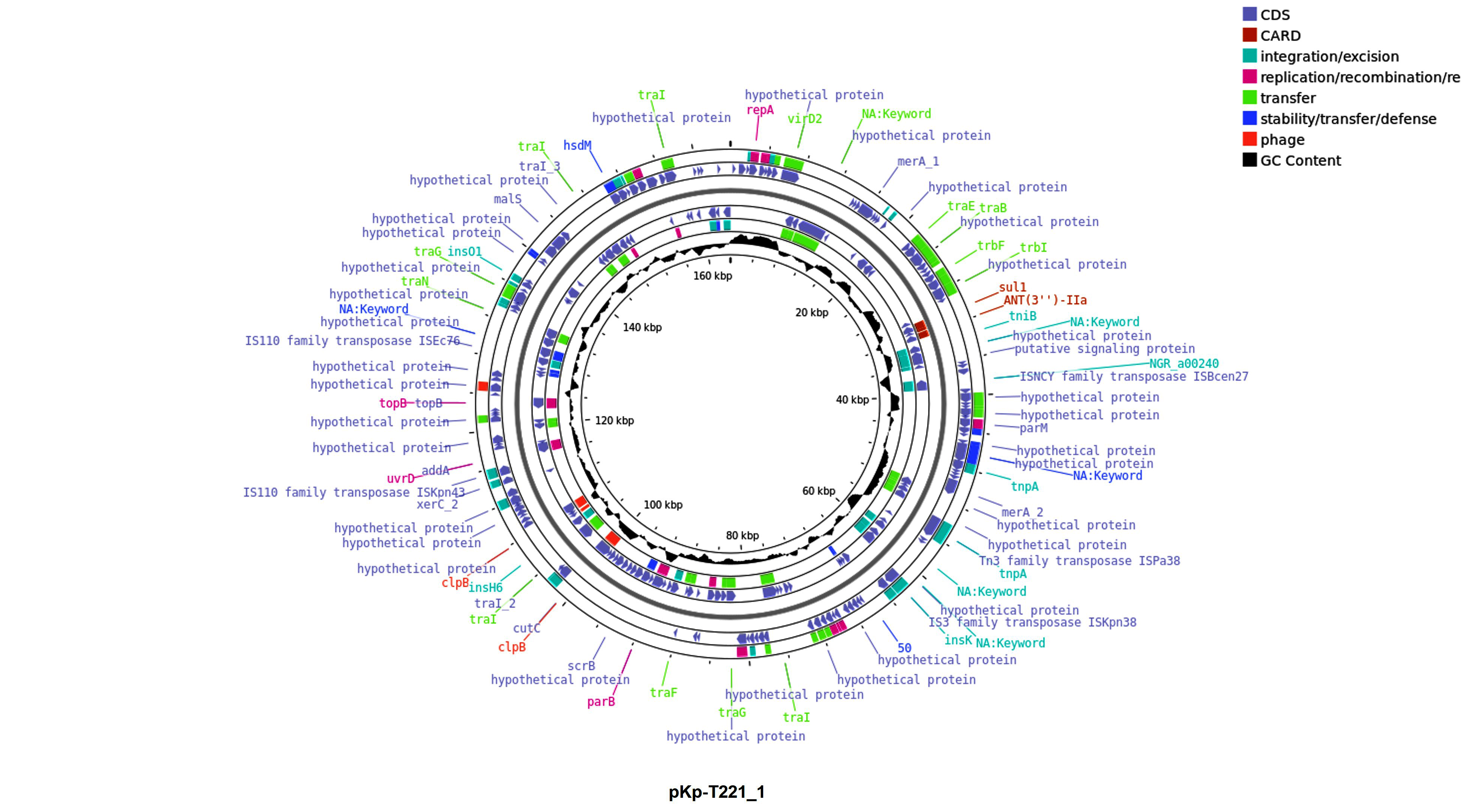

Supplement: Supplementary file 1 [file ijms-26-10910-s001.zip › Suplementary Figure_IJMS-3891215/Supplementary Figure S3_Annotated Map of the Putative Plasmid-Derived Contig Assembly pKp-T221_1_phs.tif]

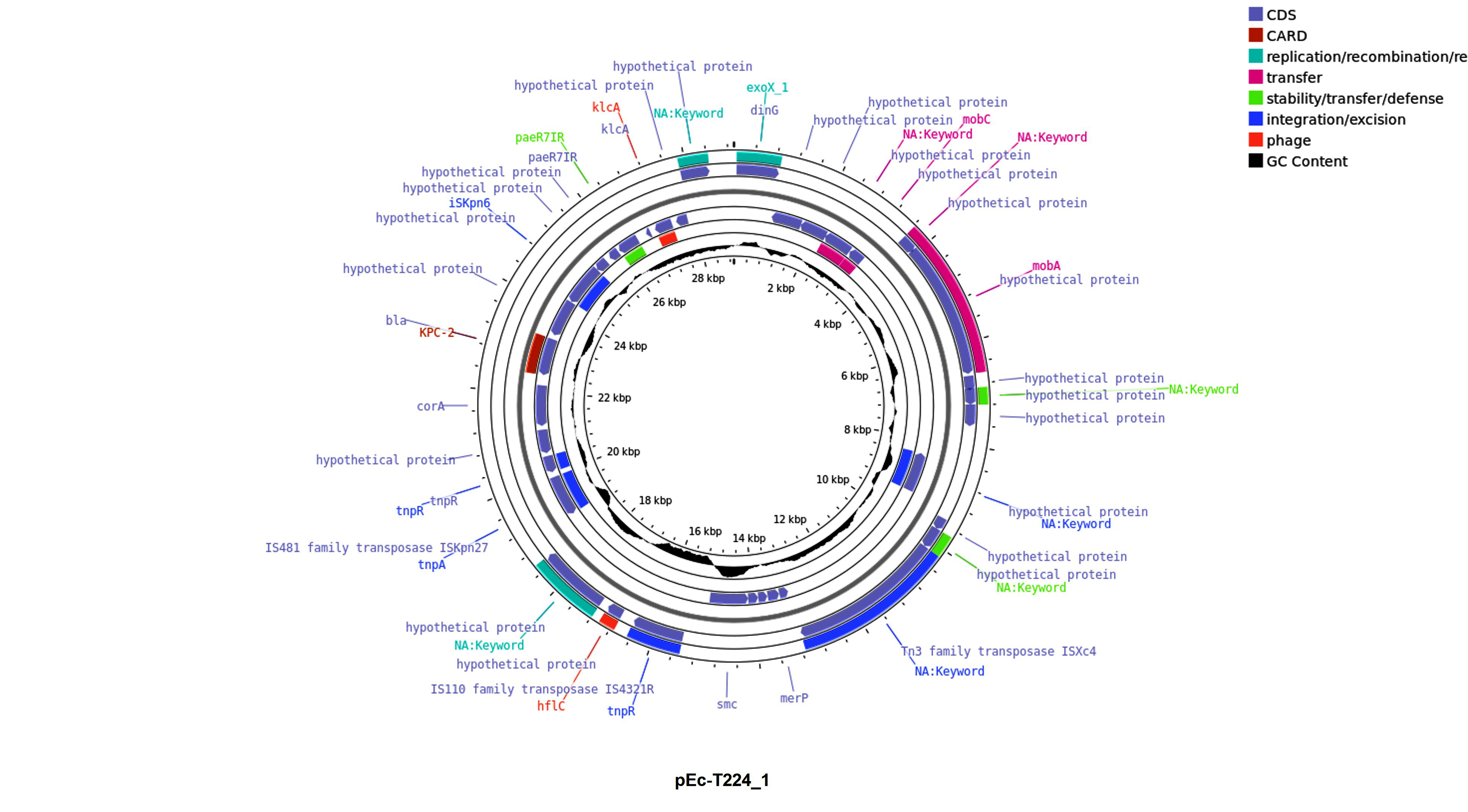

Supplement: Supplementary file 1 [file ijms-26-10910-s001.zip › Suplementary Figure_IJMS-3891215/Supplementary Figure S4_Annotated Map of the Putative Plasmid-Derived Contig Assembly pEc-T224_1_phs.tif]

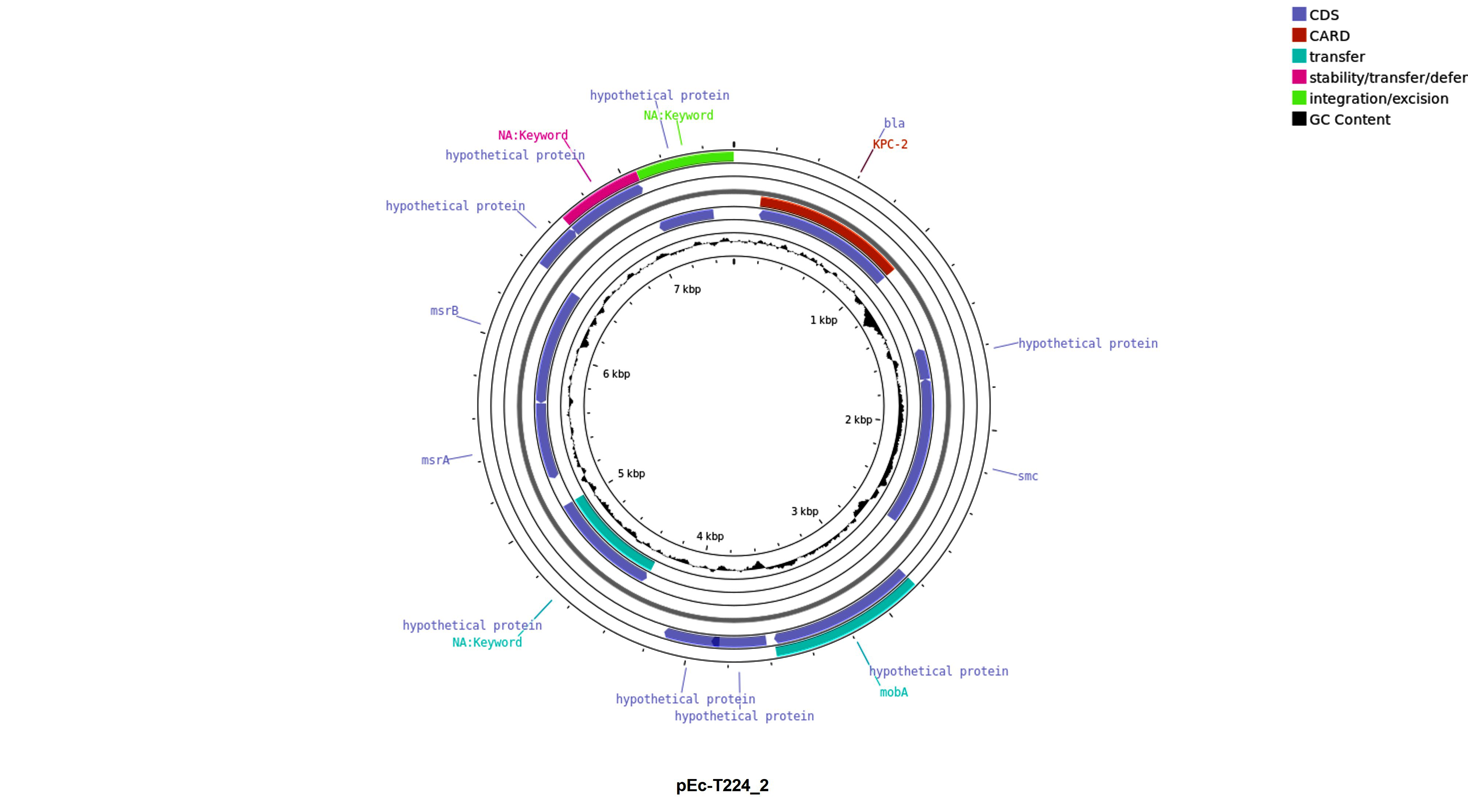

Supplement: Supplementary file 1 [file ijms-26-10910-s001.zip › Suplementary Figure_IJMS-3891215/Supplementary Figure S5_Annotated Map of the Putative Plasmid-Derived Contig Assembly pEc-T224_2_phs.tif]

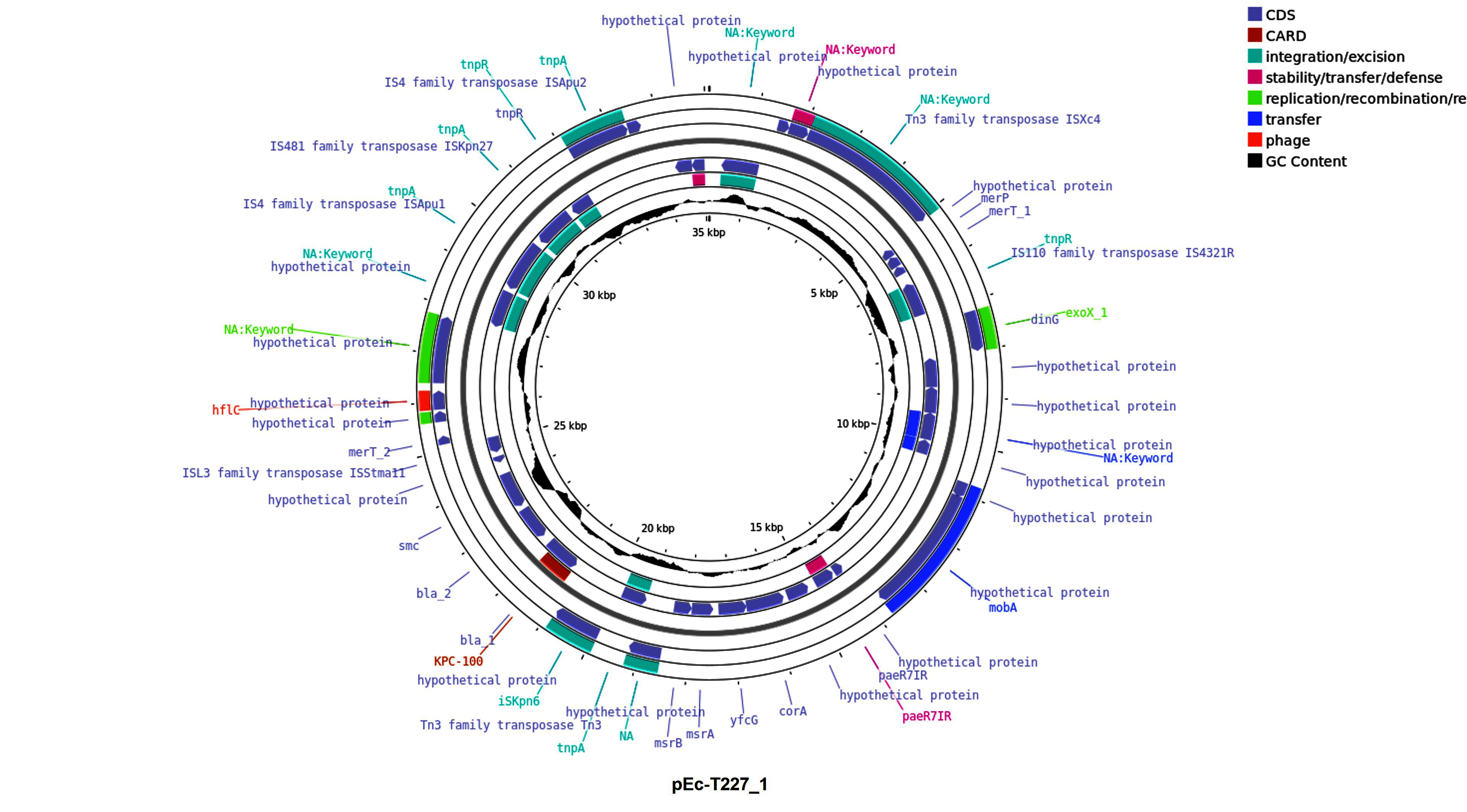

Supplement: Supplementary file 1 [file ijms-26-10910-s001.zip › Suplementary Figure_IJMS-3891215/Supplementary Figure S6_Annotated Map of the Putative Plasmid-Derived Contig Assemblyp Ec-T227_1_phs.tif]

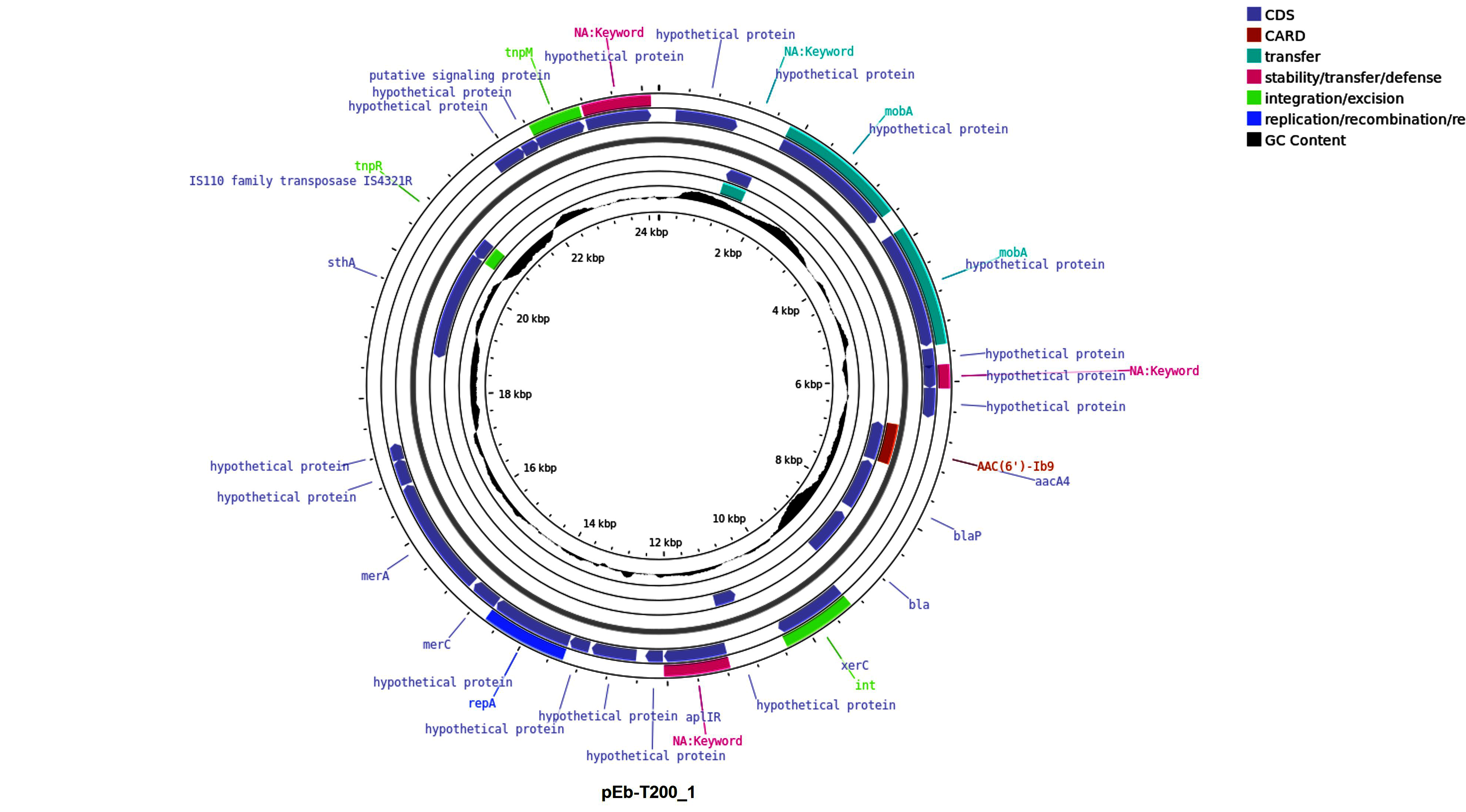

Supplement: Supplementary file 1 [file ijms-26-10910-s001.zip › Suplementary Figure_IJMS-3891215/Supplementary Figure S7_Annotated Map of the Putative Plasmid-Derived Contig Assembly pEb-T200_1_phs.tif]

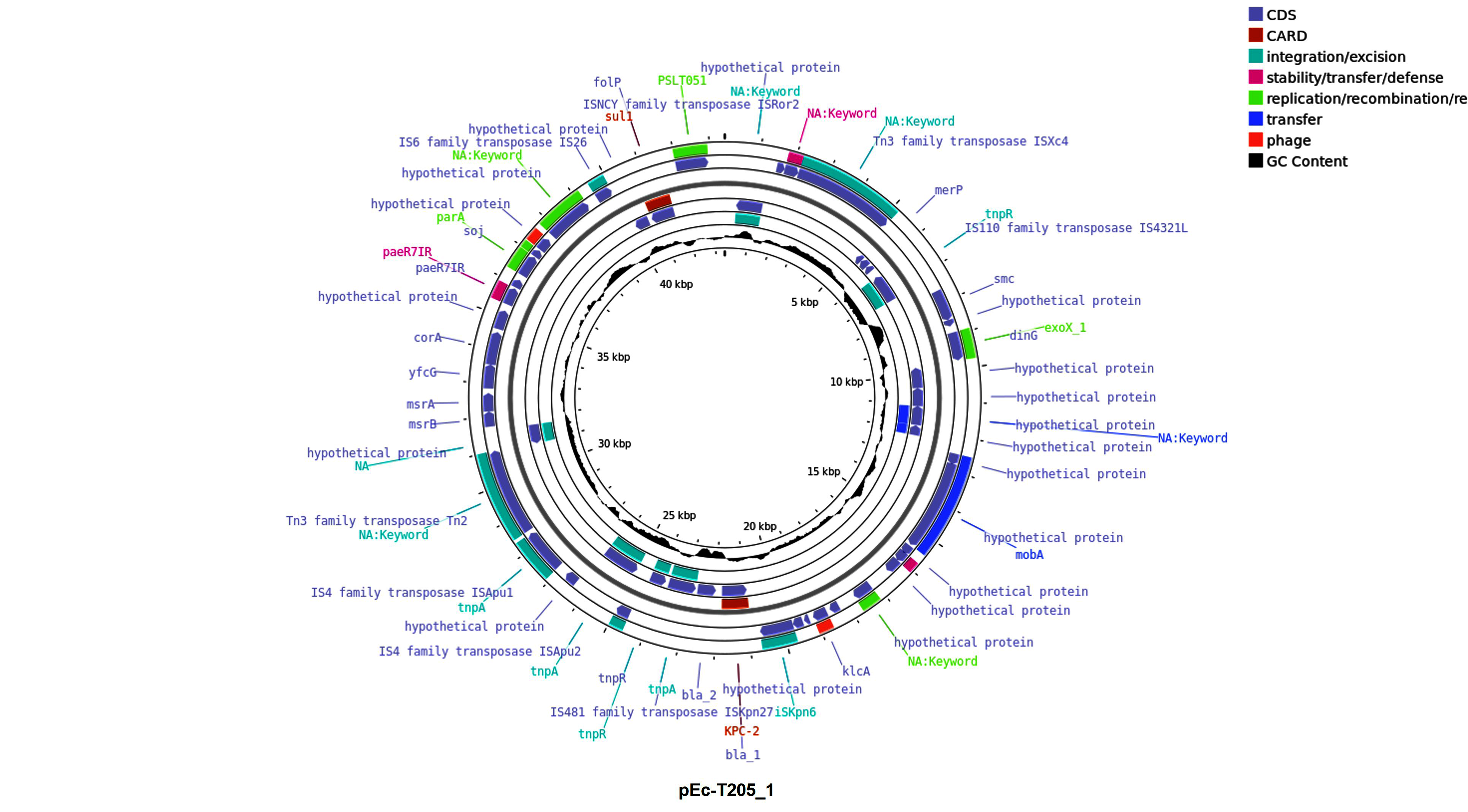

Supplement: Supplementary file 1 [file ijms-26-10910-s001.zip › Suplementary Figure_IJMS-3891215/Supplementary Figure S8_Annotated Map of the Putative Plasmid-Derived Contig Assembly pEc-T205_1_phs.tif]
